# Supplementary material for: The non-adrenergic imidazoline-1 receptor protein nischarin is a key regulator of astrocyte glutamate uptake
Source: iScience. 2022 Mar 21;25(4):104127. doi: 10.1016/j.isci.2022.104127 (PMC9010640; doi:10.1016/j.isci.2022.104127)
Supplement: Document S1. Figures S1–S — 5 [file mmc1.pdf]

iScience, Volume *25*

## Supplemental information

# The non-adrenergic imidazoline-1 receptor protein nischarin is a key regulator of astrocyte glutamate uptake

**Swati Gupta, Narges Bazargani, James Drew, Jack H. Howden, Souvik Modi, Sana Al Awabdh, Hélène Marie, David Attwell, and Josef T. Kittler**

Supplementary Figure 1, Related to Fig. 1

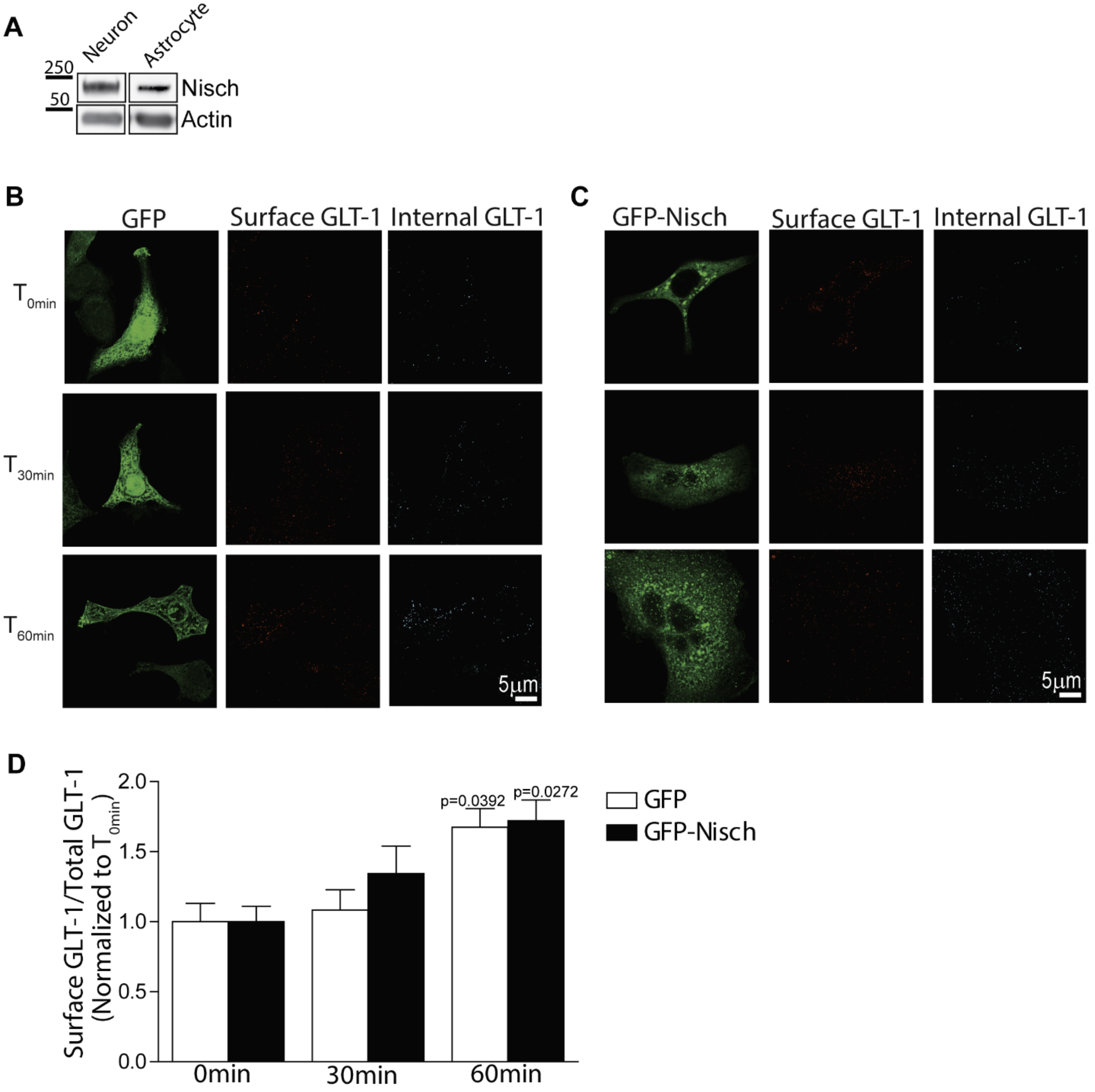


**Suppl. Figure 1, Related to Fig. 1:**

**A**) Representative western blots for endogenous Nischarin present in cortical neuron culture and pure astrocytic culture.

Nischarin does not affect GLT-1 recycling. HeLa cells co-expressing **B**) GFP and GLT-1a-HA or **C**) GFP-Nisch and GLT-1a-HA were live-labelled with anti-HA and rate of recycling was assayed using antibody feeding. **D**) No difference in recycled GLT-1 levels was observed in the two groups

at $T_{30\ min}$ and $T_{60\ min}$. At $T_{60\ min}$, both GFP control and GFP-Nisch overexpressing cells showed significant recycling of GLT-1 to the surface compared to $T_{0\ min}$, One-way ANOVA, post hoc Tukey's test ($n = 12$).

Supplementary Figure 2, Related to Fig. 2

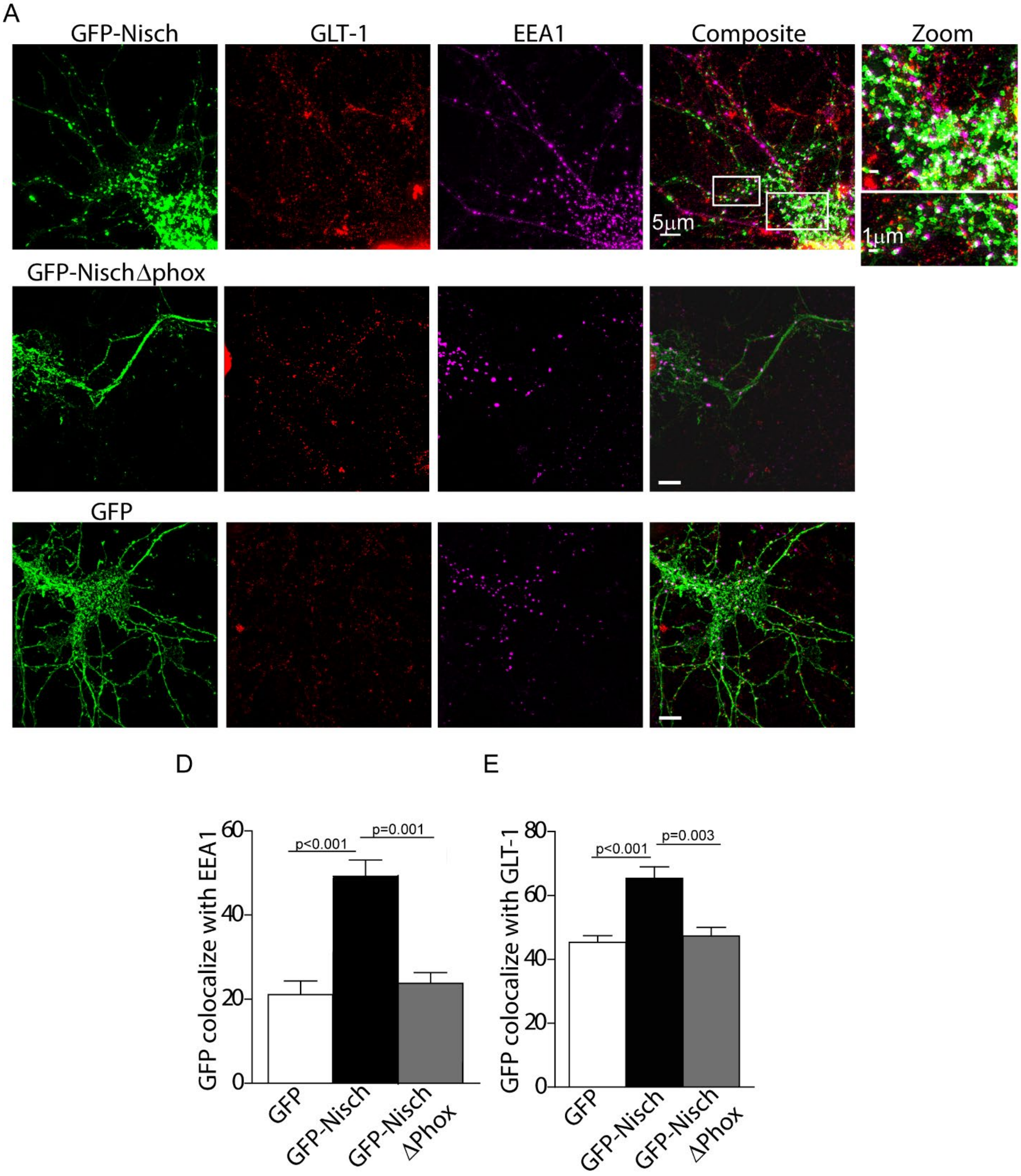


**Suppl. Figure 2, Related to Fig. 2:** Structured illumination microscopy showing **A**) GFP-Nisch or **B**) GFP-NischΔphox or **C**) GFP and endogenous GLT-1 (red) and EEA1 (magenta) in astrocytes of DIV14 hippocampal culture.

**D**) Significant colocalization was observed between GFP-Nisch and the endogenous endosomal marker, EEA1 in comparison to the GFP-NischΔphox mutant and GFP control. One-way ANOVA, Kruskal Wallis test with Dunn's multiple comparison test (*n* = 18-24 cells).

**E**) Significant colocalization was observed between GFP-Nisch and endogenous GLT-1 in comparison to GFP-NischΔphox mutant and GFP control. One-way ANOVA, post hoc Tukey's test (*n* = 15-20 cells).

Supplementary Figure 3, Related to Figs. 3 and 4

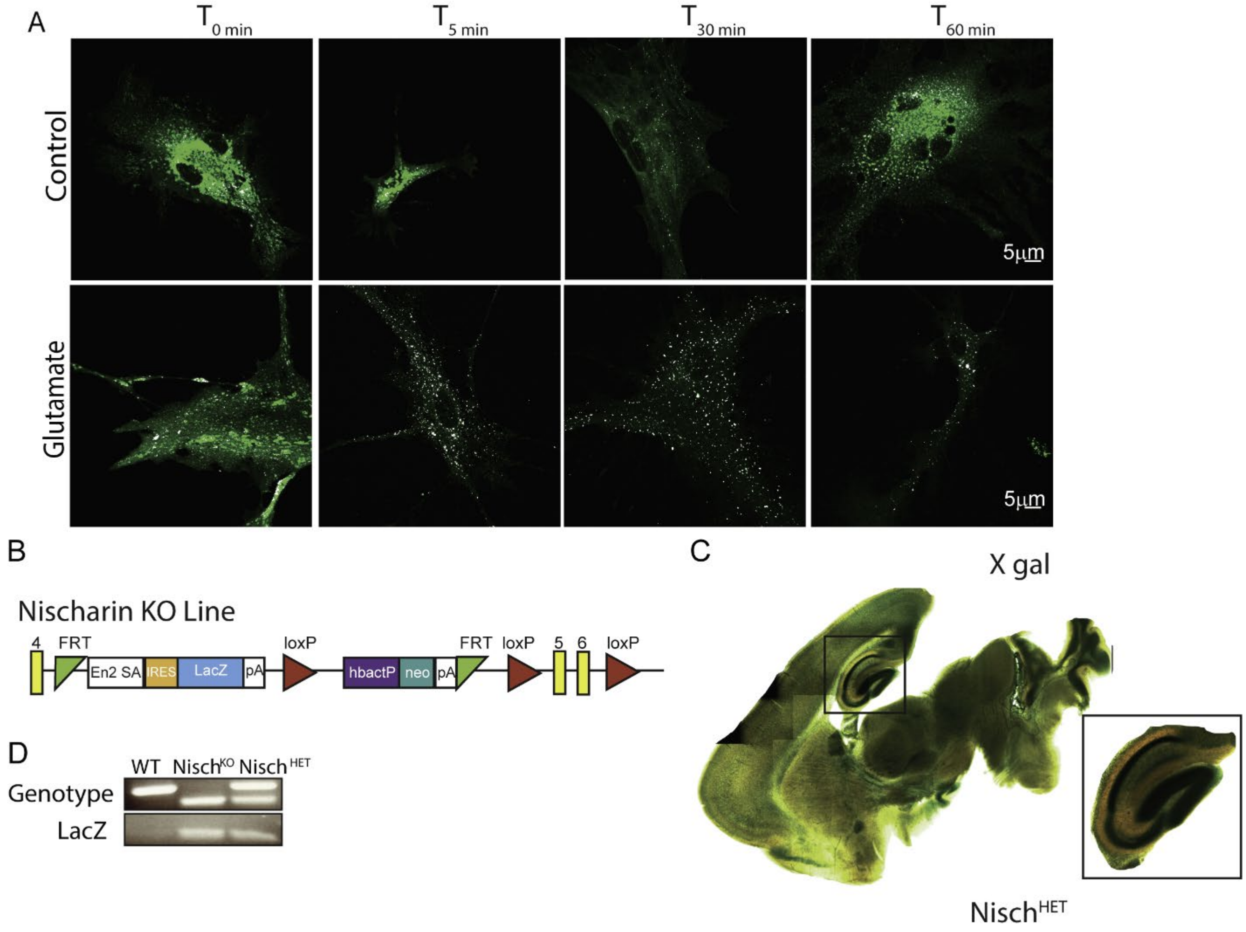


**Suppl. Figure 3, Related to Fig. 3 and 4:**

**A**) Confocal images of GFP-Nisch and GLT-1aBBS expressing astrocytes treated with 100µM glutamate for 0, 5, 30 and 60 min showed increased co-localization (shown in white) between GFP-Nisch (green) and GLT-1 compared to untreated controls.

**B**) The *Nisch* transgenic line was generated following the knockout-First strategy applied to the C57BL/6N Taconic strain. A L1L2_Bact_P cassette encoding an engrailed1 splice acceptor sequence, a LacZ reporter and a neomycin resistance gene was inserted between exons 4 and 5, disrupting *Nisch* transcription.

**C**) Xgal staining (dark blue) of sagittal brain section of a Nisch$^{HET}$ animal demonstrates widespread Nischarin expression throughout the brain. Inset, hippocampus shows enriched Nischarin expression.

**D**) PCR analysis on DNA extracted from E16 WT, $Nisch^{KO}$ and $Nisch^{HET}$ embryos shows successful recognition of the WT and *Nisch* deletion alleles.

Supplementary Figure 4, Related to Fig. 3

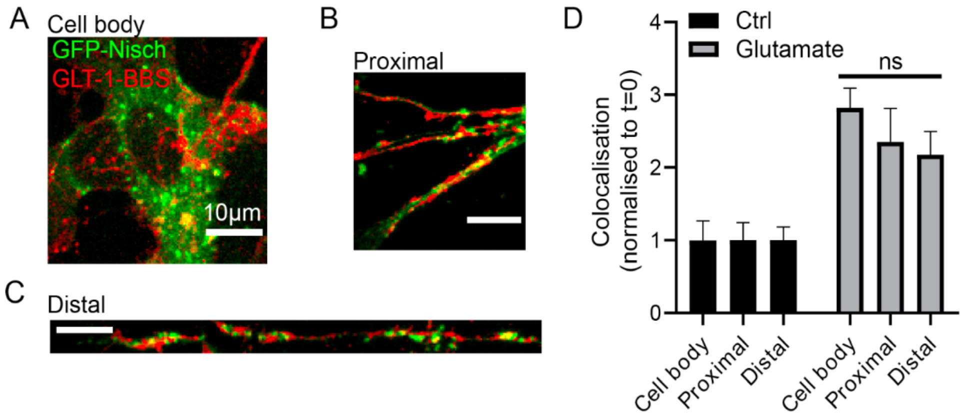


| Tukey's multiple comparisons test | Mean Diff. | 95.00% CI of diff. | Significant? | Summary | Adjusted P Value |
|---|---|---|---|---|---|
| Cell body vs. Proximal | 0.4712 | -0.5475 to 1.490 | No | ns | 0.5035 |
| Cell body vs. Distal | 0.6477 | -0.4040 to 1.699 | No | ns | 0.3018 |
| Proximal vs. Distal | 0.1764 | -0.9009 to 1.254 | No | ns | 0.9162 |

**Suppl. Figure 4, Related to Fig. 3:**

Example of GFP-Nisch and GLT-1aBBS-BTX-555 labelling in the **A**) astrocyte cell body, **B**) proximal processes and **C**) distal processes. Yellow puncta represent co-localization. Scale bar = 10µm. Proximal processes were defined as the 50% of process area closest to the cell body and the remaining 50% were defined as distal. **D**) No significant differences were observed in the sub-cellular distribution of the co-localizing puncta under control or glutamate treatment (100µM, 60min), One-way ANOVA, *post hoc* Tukey's test (n ≥10 cells and >3 preparations per treatment).

Supplementary Figure 5, Related to Fig. 4

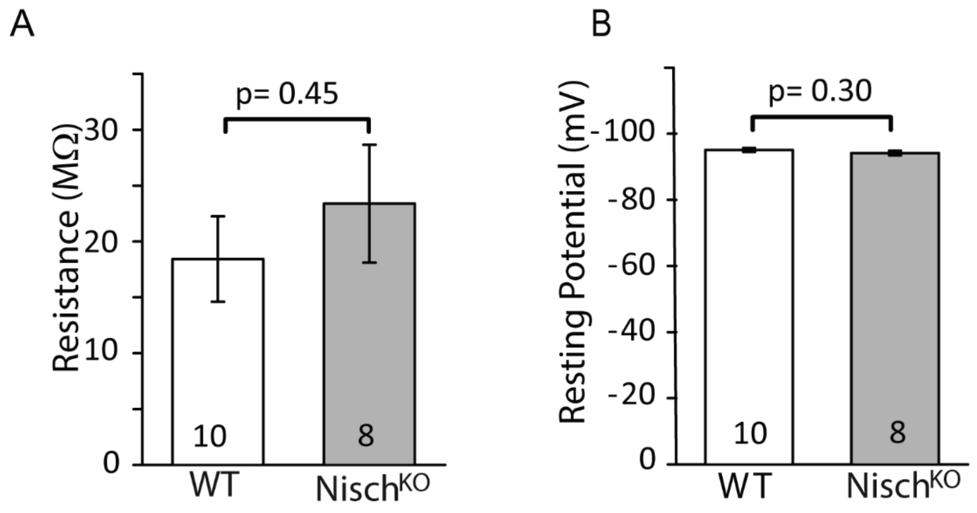


**Suppl. Figure 5, Related to Fig. 4:**

**A & B**) Astrocytes from WT and Nisch$^{KO}$ hippocampal tissue cultures showed a similar membrane resistance (**A**), and resting potential (**B**).
